# Distribution of inflammatory phenotypes among patients with asthma in Jilin Province, China: a cross-sectional study

**DOI:** 10.1186/s12890-021-01722-0

**Published:** 2021-11-12

**Authors:** Bingqing Shi, Wei Li, Hongna Dong, Mengting Xu, Yuqiu Hao, Peng Gao

**Affiliations:** Department of Respiratory Medicine, The Second Hospital of Jilin University, Changchun, 130041 Jilin China

**Keywords:** Asthma, Inflammatory cell phenotypes, Induced sputum, Uncontrolled asthma, Severe asthma

## Abstract

**Background:**

The inflammatory phenotypes of asthma predict the treatment response and prognosis. The phenotype distributions differ depending on the geographical region. This study aimed to assess the distribution of different inflammatory phenotypes among asthma patients in Jilin Province, China.

**Methods:**

A total of 255 patients with asthma were recruited from Jilin Province, China for this cross-sectional study. Each patient underwent sputum induction following clinical assessment and peripheral blood collection. Inflammatory phenotypes were classified according to the inflammatory cell counts in the sputum.

**Results:**

Paucigranulocytic asthma (PGA) was the most common inflammatory phenotype (52.2%), followed by eosinophilic asthma (EA, 38.3%), mixed granulocytic asthma (MGA, 5.2%), and neutrophilic asthma (NA, 4.3%). NA was more common among patients over 45 years old and those who were treated with higher doses of inhaled corticosteroids (ICS), but was less common following antibiotics treatment (*p* < 0.05). The proportion of patients with EA decreased as the ICS treatment dose and time increased (*p* = 0.038). Patients with uncontrolled asthma had higher numbers of sputum eosinophils and neutrophils (*p* < 0.05). Patients with severe asthma had a higher percentage of sputum neutrophils (*p* < 0.05). A greater proportion of patients with NA had severe asthma (60%) compared to those with EA (18.2%) (*p* = 0.016).

**Conclusions:**

The most common asthma inflammatory cell phenotype in Jilin Province, China is PGA, followed by EA, MGA, and NA. The low prevalence of NA in Jilin Province compared to other countries and also other regions in China might be due to excessive antibiotic use and irregular ICS treatment in this region.

## Background

Asthma is a complex and heterogeneous disease. Asthmatic airway inflammation is characterized by the infiltration of different types of inflammatory cells (neutrophils, eosinophils, T lymphocytes, etc.) in the airway tissue and the secretion of a variety of inflammatory mediators and cytokines. This leads to airway hyperresponsiveness and reversible airflow limitation [1, 2]. Based on the different types of inflammatory cells that dominate in the induced sputum, asthma can be divided into four inflammatory phenotypes: eosinophilic asthma (EA), neutrophilic asthma (NA), mixed granulocytic asthma (MGA), and paucigranulocytic asthma (PGA) [3].

EA has long been considered the most “typical” type of asthmatic airway inflammation, characterized by the infiltration of eosinophils as well as IgE-mediated mast cell activation [2]. EA generally presents with mild inflammation and good responsiveness to treatment with glucocorticoids [4]. Existing epidemiological evidence indeed shows that EA is the most common inflammatory phenotype in many regions [5, 6], but NA is more common in some regions [7, 8]. Approximately half of the patients with mild-to-moderate or refractory asthma experience persistent non-eosinophilic inflammation [9, 10]. Unlike EA patients, patients with NA respond poorly to glucocorticoid treatment even when their condition is only mild to moderate [9], and more easily develop severe asthma or refractory asthma, which is characterized by persistent airway limitation [11]. It is therefore important that patients with asthma receive individualized treatment according to their specific inflammatory phenotype.

Jilin Province in northeast China is a relatively low economically developed region with a cold climate and heavy air pollution [12–14]. A study showed that the prevalence of asthma in Northeast China is 1.69%, second only to the 2.3% in South-west China, and significantly higher than other areas in China [15]. Furthermore, patients diagnosed with asthma in Northeast China have poorer control of the disease [16]. We hypothesized that these observations might be due to differences in the distribution of inflammatory phenotypes among asthma patients in this region. To the best of our knowledge, there have been very few reports (e.g., the study by Gai et al. [7]) on the distributions of different inflammatory phenotypes of asthma throughout China. The present study therefore aimed to characterize the distribution of asthma inflammatory phenotypes in Jilin province, China, to facilitate personalized asthma treatment and management.

## Methods

### Study patients and design

Patients diagnosed with asthma were recruited from the Department of Respiratory Medicine at the Second Hospital of Jilin University for this cross-sectional study. The hospital also contains the Jilin Provincial Institute of Respiratory Diseases and is a regional center that serves patients from the entire Jilin province as well as a research center.

The patient inclusion criteria were as follows: age ≥ 18 years; diagnosis of asthma in accordance with the 2012 Global Initiative for Asthma (GINA) criteria [17]; and stable condition without acute exacerbation. The patient exclusion criteria were as follows: pregnancy; cognitive impairment; malignancy, type 2 diabetes, active tuberculosis, autoimmune diseases, kidney and liver failure, heart failure, cerebrovascular diseases, chronic obstructive pulmonary disease (COPD) (except for asthma and COPD overlap [ACO], or pulmonary diseases (except for asthma). Smoking history was not an inclusion criterion. Age- and sex-matched healthy volunteers ≥ 18 years old with no clinical signs or history of asthma or other autoimmune diseases were recruited as healthy controls.

Basic clinical information, such as smoking history, asthma treatment, and body mass index (BMI) were collected for all participants. The participants underwent lung function measurements and their asthma control and quality of life were assessed using standardized validated questionnaires. The study protocol was approved by the ethics committee of the Second Hospital of Jilin University (2016-34), and all patients provided informed consent to participate.

### Lung function measurements

All participants performed three peak flow maneuvers to ensure the correct technique was used. The fraction of exhaled nitric oxide (FENO) was measured using the Nano Coulomb Breath Analyzer (Sunvou-CA2122) following the European Respiratory Society/American Thoracic Society (ERS/ATS) guidelines [18]. Spirometry was performed using a rolling seal spirometer in accordance with the ERS/ATS guidelines [19] and the bronchodilator response was assessed 15 min after the inhalation of 400 µg of albuterol.

### Asthma control and quality of life

All enrolled patients completed the following standardized validated questionnaires: six-item Asthma Control Questionnaire (ACQ6) [20], Asthma Control Test Questionnaire (ACT) [21], Asthma Quality of Life Questionnaire (AQLQ) [22], and Hospital Anxiety and Depression scale (HADS) [23].

Uncontrolled asthma and severe asthma were defined according to the 2019 GINA guidelines [24]. Uncontrolled asthma fulfills one or two of the following criteria: (1) poor symptom control (frequent symptoms or reliever use, activity limited by asthma, night waking due to asthma); (2) frequent exacerbations (≥ 2/year) requiring oral corticosteroids or serious exacerbations (≥ 1/year) requiring hospitalization. Severe asthma refers to uncontrolled asthma despite adherence to the maximal optimized therapy or asthma that worsens when high-dose treatment is reduced. Low, medium, and high doses of inhaled corticosteroids (ICS) were defined as a daily dose of budesonide (or equivalent) of 200–400 μg, 400–800 μg, and > 800 μg, respectively [25]. Atopy was defined as the presence of at least one common aeroallergen (e.g., cat, dog, house dust mites, grass pollen, tree pollen, and a mixture of molds) [26].

### Induced sputum collection and inflammatory phenotype classification

Induced sputum was collected and processed using validated methods [27, 28]. Briefly, all patients underwent sputum induction with 4.5% hypertonic saline for 15 min. The sputum samples obtained were treated with dithiothreitol and the cells were resuspended in phosphate-buffered saline. The suspension (60 μm) was then filtered and the total leukocyte cell count (inflammatory cell count × 0.02/quadrant, TCC) was evaluated. Single-cell smears were prepared using cytospin and stained with May-Grunwald Giemsa. Differential counts of the inflammatory cell eosinophils, neutrophils, macrophages, and lymphocytes were manually obtained from 400 non-squamous cells under a light microscope.

The presence of eosinophilic inflammation was defined as ≥ 3% eosinophils in the induced sputum and the presence of neutrophilic inflammation was defined as ≥ 61% neutrophils in the induced sputum, based on published criteria [3]. Accordingly, the following four inflammatory phenotypes were identified: EA (sputum eosinophils ≥ 3%), NA (sputum neutrophils ≥ 61%), MGA (sputum eosinophils ≥ 3% and neutrophils ≥ 61%), and PGA (sputum eosinophils < 3% and neutrophils < 61%).

### Statistical analysis

IBM SPSS Statistics, version 25.0, was used for statistical analysis in this study. Data are expressed as the mean ± standard deviation (SD) or median (interquartile range). Subgroups were compared using one-way analysis of variance (ANOVA) with a least significant difference test or a Kruskal–Wallis test, accompanied by Bonferroni correction. Categorical variables are presented as proportions of observations, and were analyzed using the chi-squared test. Spearman correlation coefficients were used to identify correlations between the clinical characteristics of patients. Differences with *p* < 0.05 were considered statistically significant.

## Results

### Demographic, functional, and inflammatory characteristics of patients with asthma vs healthy person

In total, 255 patients with asthma and 22 healthy volunteers were enrolled in this study (Table 1). Basic demographic variables, including average age, BMI, male/female ratio, and percentage of smokers were not significantly different between the healthy volunteers and asthma patients (*p* > 0.05). Compared with the healthy controls, the predicted forced expiratory volume in 1 s (FEV1) and FEV1/Forced vital capacity (FVC) in asthma patients were significantly lower (*p* < 0.05). The success rates of sputum induction in both asthmatic patients and healthy volunteers were higher than 80%. The average percentage of sputum eosinophils in the asthma group was significantly higher than that in the healthy control group (*p* < 0.001), but the average percentage of sputum neutrophils was lower (*p* = 0.03) (Table 1). The percentage of sputum macrophages in the control group was not significantly different from that in the asthma group, but was significantly lower than the percentage in the PGA group (*p* = 0.03).

**Table 1:** Demographic, clinical, and inflammatory characteristics in patients with asthma and healthy person

Characteristic	Control	Asthma	*p* value
N	22	255	–
Age, years	54 ± 14	51 ± 14	NS
Sex (M/F)	11/11	116/140	NS
BMI (kg/m^2^)	23.7 ± 2.6	23.7 ± 3.4	NS
Ex-smokers (n) (pack-years)	10 (5.8 (4.4, 6.6))	104 (20.3 (9.8, 37.0))	NS
Pre-FVC	3.3 (2.7, 4.0)	2.9 (2.2, 3.6)	< 0.001
Pre-FEV1	2.7 (2.2, 3.2)	1.7 (1.1, 2.4)	NS
FEV1/FVC (%)	79.2 ± 6.2	60.1 ± 13.8	< 0.001
FEV1% predicted	78 (74.6, 82.4)	64.3 (41.2, 82.9)	0.002
Sputum TCC (10^6^/mL)	2.4 (1.4, 5.1)	3 (1.8, 5.8)	NS
Sputum eosinophils (%)	0.1 (0, 0.4)	2 (0.2, 9.9)	< 0.001
Sputum neutrophils (%)	9.6 (4.1, 22.1)	1.6 (0.2, 11.6)	0.030
Sputum macrophages (%)	84 (72.4, 90.6)	81.4 (46.8, 93.9)	NS
Sputum lymphocytes (%)	2 (1, 4.2)	2.3 (0.6, 7.2)	NS

Data are presented as mean ± SD or median (range). BMI, body mass index; FEV1, forced expiratory volume in 1 s; FVC, forced vital capacity; TCC, total cell count; NS, non-significant

### Distribution of inflammatory phenotypes among patients with asthma

**Fig. 1:**
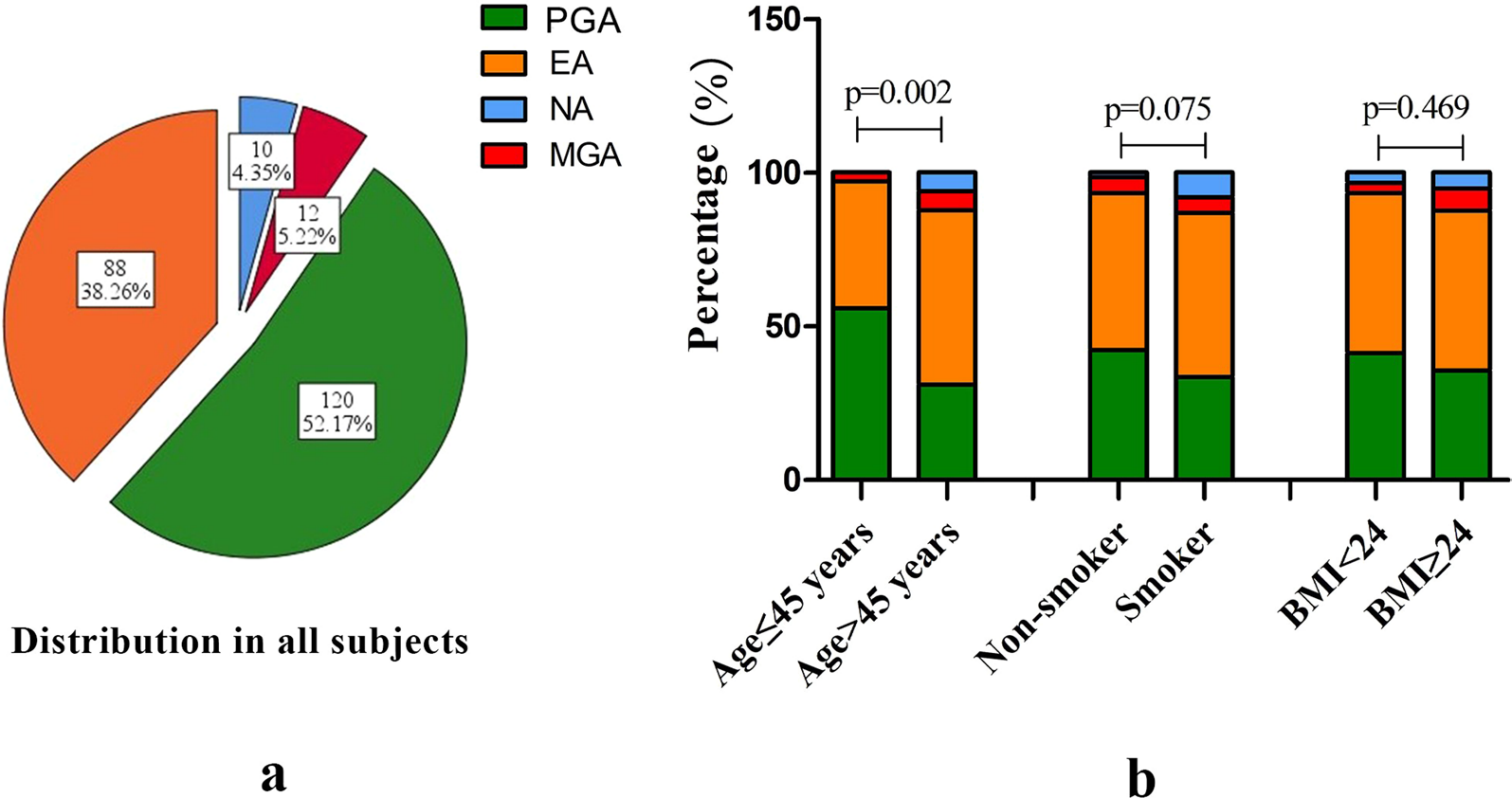
The distribution of four inflammatory phenotypes among patients with asthma and different subgroups. (**a**) The distribution of asthma inflammatory phenotype in all subjects. (**b**) Distribution characteristics of the inflammatory phenotype of asthma in different groups divided by age group, BMI classification, and smoking history. BMI, body mass index; NA, neutrophilic asthma; EA, eosinophilic asthma; PGA, paucigranulocytic asthma; MGA, mixed granulocytic asthma; ICS, inhaled corticosteroid

Among the asthma cases (Fig. 1a), 120 (52.2%) were categorized as PGA, 88 (38.3%) as EA, 12 (5.2%) as MGA, and 10 (4.3%) as NA. The prevalence of NA was significantly higher among patients > 45 years old than among those ≤ 45 years old (*p* = 0.002). EA was also significantly higher among patients > 45 years old, but to a lesser degree (Fig. 1b). NA was more common in smokers than in non-smokers, but the difference did not reach statistical significance (*p* = 0.075). There was no significant difference in the distribution of inflammatory phenotypes between patients with BMI ≥ 24 (overweight/obesity) and those with BMI < 24 (normal weight) (Fig. 1b).

**Fig. 2:**
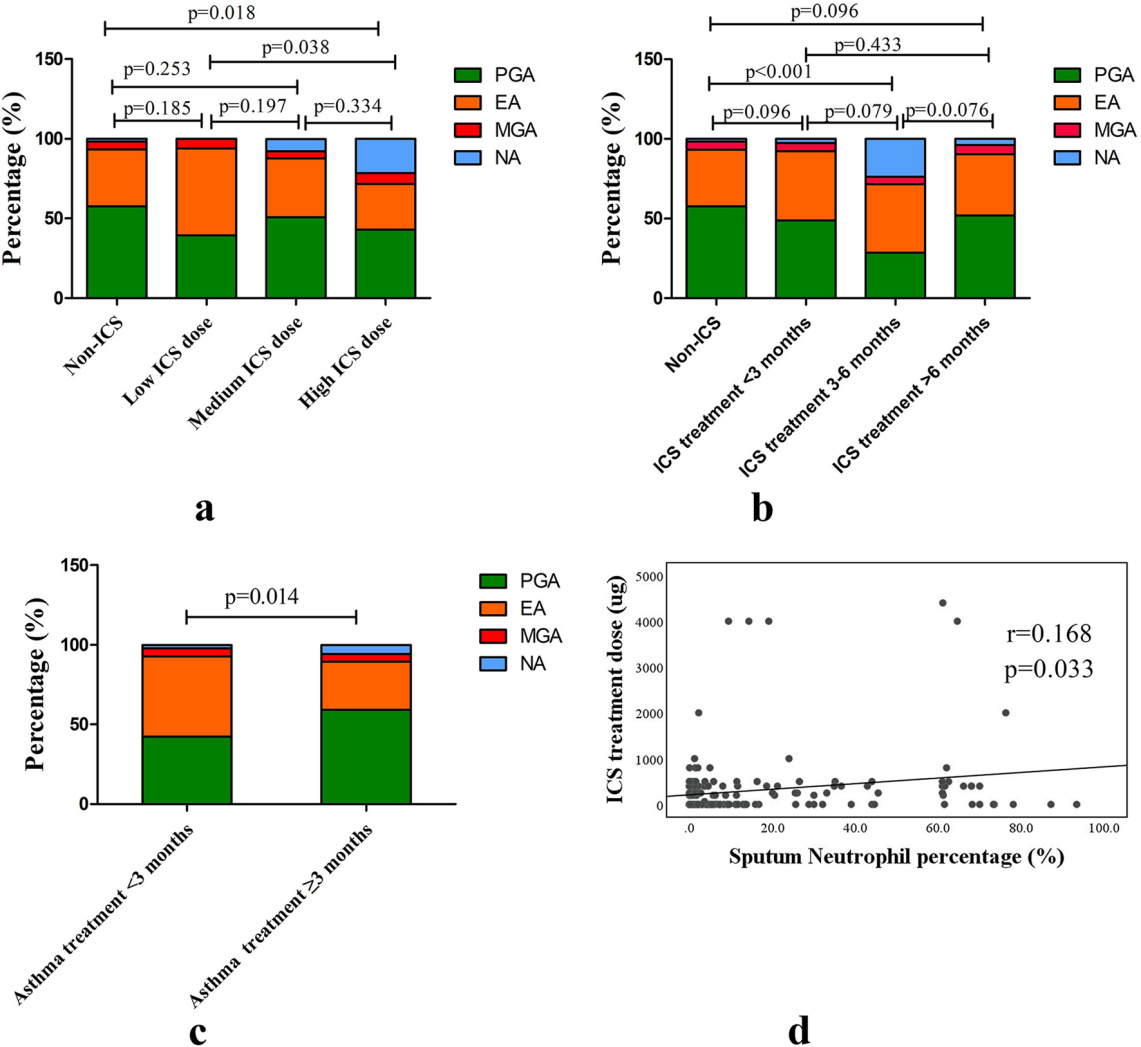
Changes in the proportions of four asthma phenotypes in different asthma treatment groups. NA, neutrophilic asthma; EA, eosinophilic asthma; PGA, paucigranulocytic asthma; MGA, mixed granulocytic asthma; ICS, inhaled corticosteroid

Among the asthma patients, 125 (48.1%) received treatment with ICS in the past year. Interestingly, the proportion of patients with NA gradually increased as the ICS dose increased, and NA was markedly more common in patients treated with ICS than in those who did not receive ICS treatment (*p* < 0.05) (Fig. 2a). Conversely, the proportion of patients with EA gradually decreased with higher ICS doses (*p* = 0.038). The prevalence of NA was highest among those who received regular ICS treatment for 3–6 months, compared with the prevalence in patients who used ICS treatment for more than 6 months or less than 3 months and those who did not receive ICS treatment. On the other hand, the proportion of patients with EA gradually decreased as the length of ICS treatment increased (*p* < 0.05) (Fig. 2b). In addition, the ICS treatment dose was positively correlated with the sputum neutrophil percentage (r = 0.168, *p* = 0.03) (Fig. 2d), but not significantly correlated with the sputum eosinophil percentage in patients with asthma (*p* > 0.05) (data not shown). Compared to patients who were treated by an asthma specialist for less than 3 months, those who underwent specialist treatment for 3 months or more had a significantly greater proportion of NA, but a lower proportion of EA (*p* = 0.014) (Fig. 2c). Furthermore, the proportion of NA among patients who did not receive treatment with antibiotics during the previous month was 4.5%, but the number dropped to 0 among those who received continuous treatment (*p* < 0.05).

### Demographic and clinical characteristics according to inflammatory phenotypes

The male/female ratio and average BMI did not differ between patients with the four inflammatory phenotypes (*p* > 0.05), but patients with NA had a higher average age and higher proportions of current smoker and ex-smoker compared with the other groups (*p* = 0.001) (Table 2). There was no atopic asthma in the NA group, which was significantly different from the findings in other groups (EA, 45%; MGA, 40%; PGA, 36%) (*p* < 0.05). Triggering factors and complications/comorbidities were not significantly different among the four groups (*p* > 0.05). The prevalence of severe asthma in patients with NA was 60%, which was significantly higher than the 18.2% among patients with EA (*p* = 0.016). Both the NA and EA groups included a higher proportion of patients who exhibited uncontrolled asthma compared to the proportion in the other groups (*p* = 0.049). In addition, in the previous year, patients with NA required the highest daily ICS dose on average, while the PGA group required the lowest (*p* = 0.015) (Table 2). The EA group had the highest average ACQ6 and HAD scores, and the lowest ACT score, but the differences between the groups were not statistically significant (*p* > 0.05). Patients with PGA showed a tendency for better lung function (FEV_1_/FVC and FEV_1_% predicted), but this difference was not statistically significant either. Patients in the EA group had higher FeNO levels compared to those in the NA and PGA groups (*p* = 0.005). Patients with EA also had greater numbers of blood eosinophils, lymphocytes, and basophils, but a lower percentage of neutrophils, compared to those with NA (*p* < 0.05).

**Table 2:** The demographic and clinical characteristics according to the inflammatory phenotype

	NA	EA	MGA	PGA	*p* value
N	10	88	12	120	< 0.001
Sex (M/F)	5/5	37/51	5/7	62/58	0.55
Age (years)	60 ± 7^#^	46 ± 14^‖^	57 ± 10	52 ± 13	0.001
BMI	24.3 ± 3.7	23.9 ± 3.5	24.3 ± 3.6	23.9 ± 3.2	NS
Smoking history					< 0.001
Never smoker, N (%)	2 (20%)^†§‡^	55 (62.5%)*	7 (58.3%)*	68 (56.7%)*	
Current smoker, N (%)	3 (30%)^†§‡^	22 (25%)*	3 (25%)*	29 (24.1%)*	
Ex-smoker, N (%)	5 (50%)^†§‡^	11 (12.5%)*	2 (16.7%)*	23 (19.2%)*	
Pack-years	9.8 (0, 50)	0 (0, 15.5)	13.5 (0, 24.3)	0 (0, 19.1)	NS
Diagnosis age (years)	54 ± 13^†^	42 ± 13*^¶^**	51 ± 10^¶^	44 ± 15^¶^	0.010
Atopy, N (%)	0 (0%)^†§‡^	22 (45%)*	2 (40%)*	21 (36%)*	NS
Triggering factors (Y/N)	9/1	81/7	9/3	99/21	NS
Complications and comorbidities (Y/N)	8/2	42/42	5/5	62/50	NS
Severe asthma, N (%)	6 (60%)**^¶#^	16 (18.2%)^‖^**	1 (8.3%)^‖#¶^	20 (16.7%)^‖^**	0.016
Uncontrolled asthma, N (%)	8 (88.9%)	69 (81%)	6 (66.7%)	70 (64.8%)	0.049
ICS daily dose	400 (400, 800)**^¶#^	200 (0, 400)^‖^	100 (0, 400)^‖^	0 (0, 400)^‖^	0.015
FEV1/FVC (%)	57.3 ± 14.1	61.5 ± 12.7	60.1 ± 16.9	62.0 ± 14.4	NS
FEV1% predicted	64.2 ± 24.7	63.4 ± 21.9	60.5 ± 30.7	71.8 ± 23.3	NS
FeNO (ppd)	30.0 (17.0, 85.0)^#^	52.5 (32.6, 99.7)^‡‖^	45.4 (26, 94.2)	31.8 (20.4, 62.1)^†^	0.005
Blood eosinophils (10^9^/L)	0.22 (0.1, 0.3)^#^	0.4 (0.2, 0.6)^‖‡^	0.20 (0.1, 0.4)	0.17 (0.1, 0.3)^†^	< 0.001
Blood eosinophils (%)	3.8 (1.3, 5.3)^#^	5.2 (2.8, 9.2)^‖‡^	2.6 (1.0, 5.7)	2.2 (1, 4.2)^†^	< 0.001
Blood neutrophils (10^9^/L)	4.7 (3.0, 9.6)	4 (3.3, 5.1)	4.5 (3.5, 6.0)	4.4 (3.3, 5.8)	NS
Blood neutrophils (%)	70 ± 11.0^‡†^	56.1 ± 10.1*^‡^**	63.6 ± 9.7^#^	60.9 ± 11.7^‖†^	< 0.001
Blood lymphocytes (10^9^/L)	1.6 (1.3, 2.1)^#^	2.4 (1.9, 2.8)^‖^	2 (1.9, 2.5)	2 (1.7, 2.6)	0.024
Blood lymphocytes (%)	24.3 ± 7.5^‡†^	31.3 ± 9.7*	26.6 ± 9.6	28.6 ± 10.3^‖^	0.04
Blood basophils (10^9^/L)	0 (0, 0.04)	0.04 (0, 0.1)**^‡^	0 (0, 0.01)^#^	0 (0, 0.05)^†^	0.009
Blood basophils (%)	0 (0, 0.7)	0.5 (0, 1.2) **^‡^	0 (0, 0.1)^#^	0 (0, 0.7)^†^	0.008
ACQ6	1.4 ± 0.9	1.8 ± 1.0	1.6 ± 1.3	1.5 ± 1.0	NS
ACT	17.8 ± 4.3	16.2 ± 4.3	17.7 ± 4.9	17.9 ± 4.8	NS
Global AQLQ	4.2 ± 2.5	5.0 ± 1.1	5.2 ± 1.4	5.1 ± 1.1	NS
HAD	1.0 (0, 10)	3.5 (0, 11)	2.5 (0, 20.5)	2.5 (0, 9)	NS

NA, neutrophilic asthma; EA, eosinophilic asthma; MGA, mixed granulocytic asthma; PGA, paucigranulocytic asthma; BMI, body mass index; ICS, inhaled corticosteroid; FEV1, forced expiratory volume in 1 s; FVC, forced vital capacity; AQLQ, Asthma Quality of Life Questionnaire; HADS, Hospital Anxiety and Depression scale; NS, non-significant

*p* < 0.01: *vs NA, ^†^vs EA, ^‡^vs PA, ^§^vs MGA. *p* < 0.05: ^‖^vs NA, ^¶^vs PA, ^#^vs EA, **vs MA

### Clinical and inflammatory characteristics of patients with uncontrolled vs. controlled asthma

There were 170 (73%) patients with uncontrolled asthma and 63 (27%) patients with controlled asthma in this study (Table 3). The male/female ratio and average age between the two groups were not significantly different (*p* > 0.05). However, the average BMI was lower among patients with uncontrolled asthma than among those with controlled asthma (*p* = 0.031), whereas the number of smokers and smoking pack-years were significantly higher (*p* < 0.05). There were higher proportions of NA and EA, but a lower proportion of PGA in the uncontrolled asthma group (*p* < 0.05). Interestingly, patients with uncontrolled asthma had higher percentages of eosinophils and neutrophils in their sputum, but a lower percentage of macrophages than those with controlled asthma (*p* < 0.05). There were no significant differences in blood eosinophil, neutrophil, or basophil counts between the two groups (*p* > 0.05), but the blood lymphocyte count was significantly higher in the uncontrolled asthma group (*p* < 0.022).

**Table 3:** Demographic, clinical, and inflammatory characteristics of patients with controlled and uncontrolled asthma

Characters	Uncontrolled	Controlled	*p* value
N	170	63	–
Age (years)	50 ± 12.7	47 ± 12.9	NS
Sex (F, %)	98 (58%)	34 (54.8%)	NS
BMI (kg/m^2^)	23.7 ± 3.3	24.9 ± 3.5	0.031
Smokers (N, %)	78 (46.2%)	18 (29%)	0.019
-Pack-years	0.8 (0, 22)	0 (0, 4.9)	0.014
NA (N, %)	8 (5.2%)	1 (1.7%)	0.020
EA (N, %)	69 (45.1%)	16 (27.1%)	< 0.001
PGA (N, %)	70 (45.8%)	39 (66.1%)	0.003
MGA (N, %)	6 (3.9%)	3 (5.1%)	0.513
Severe asthma (N, %)	45 (26.6%)	1 (1.6%)	< 0.001
Asthma diagnosis > 1 year (N, %)	103 (61.3%)	38 (61.3%)	NS
Asthma specialist treatment > 3 m (N, %)	86 (51.2%)	41 (67.2%)	0.031
ICS treatment (N, %)	80 (47.1%)	37 (58.7%)	0.013
ICS treatment 3–6 m (N, %)	10 (5.9%)	13 (21%)	0.008
ICS + LABA (N, %)	61 (36.1%)	34 (54.8%)	0.010
ICS dose (μg)	0 (0, 400)	250 (0, 400)	NS
SABA treatment	33 (19.5%)	4 (6.5%)	0.016
Exacerbation (N, %)	58 (34.3%)	8 (12.9%)	0.001
Persistent cough (N, %)	62 (36.9%)	7 (11.3%)	< 0.001
COPD (N, %)	13 (7.9%)	0 (0%)	0.033
Pre-FEV1/FVC%	59.8 ± 12.8	65 ± 14.8	0.003
FEV1% predicted	65.7 ± 25.2	67.4 ± 26.7	NS
Post- FEV1/FVC%	63.5 ± 13.2	68.3 ± 14.8	0.010
Global AQLQ	4.7 ± 1.2	6.1 ± 0.9	< 0.001
HADS	5 (0, 11)	0 (0, 4.7)	< 0.001
White blood cells (10^9^/L)	8.0 ± 2.5	7.2 ± 1.9	0.026
Blood eosinophils (10^9^/L)	0.22 (0.1, 0.5)	0.2 (0.1, 0.5)	NS
Blood neutrophils (10^9^/L)	4.3 (3.4, 5.4)	3.7 (3.1, 5.8)	NS
Blood lymphocytes (10^9^/L)	2.3 ± 0.9	2.1 ± 0.7	0.022
Blood basophils (10^9^/L)	0 (0, 0.1)	0 (0, 0.8)	NS
Sputum TCC (10^6^/mL)	2.9 (1.8, 5.4)	3.3 (2.1, 7.6)	NS
Sputum macrophages (%)	82 (49.7, 93)	92.6 (73.7, 97.5)	0.010
Sputum neutrophils (%)	2 (0.2, 11.2)	0.4 (0, 1.7)	0.012
Sputum eosinophils (%)	2.9 (0.5, 9.4)	1.3 (0, 3.9)	0.003
Sputum lymphocytes (%)	1.6 (0.5, 4.8)	1.7 (0.1, 2.9)	NS

BMI, body mass index; NA, neutrophilic asthma; EA, eosinophilic asthma; PGA, paucigranulocytic asthma; MGA, mixed granulocytic asthma; ICS, inhaled corticosteroid; LABA, long-acting β2-adrenergic receptor agonists; SABA, short-acting β2 receptor agonist; COPD, chronic obstructive pulmonary disease; FEV1, forced expiratory volume in 1 s; FVC, forced vital capacity; AQLQ, Asthma Quality of Life Questionnaire; HADS, Hospital Anxiety and Depression scale; TCC, total cell count; NS: non-significant

There were 45 (26.6%) cases of severe asthma in the uncontrolled asthma group, but only one (1.6%) in the controlled asthma group (*p* < 0.001). Although the predicted FEV1% was similar in both groups, patients with uncontrolled asthma had distinctly lower pre-FEV1/FVC% and post-FEV1/FVC% compared to those with controlled asthma (*p* < 0.05). In addition, the symptoms, activity, emotion, and environment assessed with the global AQLQ score were all significantly lower in the uncontrolled group (*p* < 0.001). Furthermore, the HAD scores of patients with uncontrolled asthma were significantly higher (*p* < 0.001). Compared with the controlled asthma group, more patients in the uncontrolled asthma group suffered from chronic obstructive pulmonary disease (COPD), persistent cough, and exacerbation (*p* < 0.005), but the frequency of other complications and comorbidities (such as rhinitis, nasal polyps, etc.) was similar between the two groups (*p* > 0.05). The proportions of patients who received their asthma diagnosis more than one year earlier were not significantly different between the two groups (*p* > 0.05). However, the proportions of patients receiving ICS or ICS + Long-acting β2-adrenergic receptor agonist (LABA) treatment was significantly lower in the uncontrolled asthma group (*p* < 0.05), whereas the proportion that persistently used a short-acting β2 receptor agonist (SABA) was higher (*p* = 0.016). Patients with uncontrolled asthma were treated with lower ICS doses, but the difference was not statistically significant (*p* = 0.191). A significantly higher proportion of patients were treated by a specialist for more than 3 months in the uncontrolled asthma group compared to the corresponding proportion in the controlled group (*p* = 0.031).

### Clinical and inflammatory characteristics of patients with severe and non-severe asthma

A relatively small proportion of the asthma patients included in this study had severe asthma (19.1%) (Table 4). There was no difference in the female/male ratio, average BMI, and smoking history between the severe and non-severe asthma groups, but the average age was higher in the severe asthma group (*p* < 0.001). The severe asthma group included a significantly higher proportion of patients with NA, but a lower proportion of those with PGA (*p* < 0.001). Compared with the non-severe asthma group, patients with severe asthma had slightly higher percentages of neutrophils and eosinophils in the sputum, but the difference was not statistically significant (*p* > 0.05). The inflammatory cells in the differential blood count were not significantly different between the two groups (*p* > 0.05).

**Table 4:** Demographic, clinical, and inflammatory characteristics of patients with severe/non-severe asthma

Characters	Severe asthma	Non-severe asthma	*p* value
N	49	207	–
Age (years)	54 ± 10	47 ± 13	< 0.001
Sex (F, %)	28 (57.1%)	112 (54.1%)	NS
BMI (kg/m^2^)	23.9 ± 3.8	24 ± 3.3	NS
Smokers (N, %)	19 (38.8%)	85 (41.1%)	NS
NA (N, %)	6 (14%)	4 (2.1%)	< 0.001
EA (N, %)	16 (37.2%)	73 (38.6%)	0.512
PGA (N, %)	20 (46.5%)	101 (53.4%)	< 0.001
MGA (N, %)	1 (2.3%)	11 (5.8%)	0.006
Asthma diagnosis > 1 year (N, %)	41 (83.7%)	108 (56.5%)	< 0.001
Asthma specialist treatment > 3 m (N, %)	42 (85.7%)	93 (48.9%)	< 0.001
Allergic conjunctivitis (N, %)	4 (8.3%)	21 (11.1%)	0.049
ICS dose (μg)	500 (400, 500)	0 (0, 250)	< 0.001
ICS + LABA (N, %)	38 (77.6%)	58 (28%)	< 0.001
Exacerbation (N, %)	37 (75.5%)	66 (34.6%)	< 0.001
Pre-FEV1/FVC%	54.2 ± 12.3	63 ± 13.3	< 0.001
FEV1% predicted	55.6 ± 20.6	68.9 ± 26	0.002
Post- FEV1/FVC%	57.6 ± 12.3	66.6 ± 13.6	< 0.001
Global AQLQ	4.5 ± 1.5	5.2 ± 1.1	0.020
ACT scores	15.4 ± 4.2	17.6 ± 4.6	0.005
≤ 15	21 (45.7%)	67 (36.2%)	0.012
16–20	18 (39.1%)	50 (27%)	0.012
21–25	7 (15.2%)	68 (36.8%)	0.012
HAD	3 (0, 11)	3 (0, 9)	NS
Blood eosinophils (10^9^/L)	0.2 (0.1, 0.5)	0.24 (0.3, 0.6)	NS
Blood neutrophils (10^9^/L)	3.5 (3, 5.4)	4.3 (3.4, 5.5)	NS
Blood lymphocytes (10^9^/L)	2.2 (1.8, 2.8)	2.1 (1.8, 2.6)	NS
Blood basophils, (10^9^/L)	0 (0, 0.04)	0 (0, 0.1)	0.044
Sputum TCC (10^6^/mL)	2.9 (1.8, 5.4)	3.2 (1.8, 5.7)	NS
Sputum macrophages (%)	82.6 (49.1, 93)	85.1 (57.5, 95.6)	NS
Sputum neutrophils (%)	2.2 (0, 16.3)	1 (0, 6.7)	NS
Sputum eosinophils (%)	2.9 (0.5, 9.9)	2.2 (0.2, 7.5)	NS
Sputum lymphocytes (%)	1.4 (0, 6.5)	1.7 (0.5, 4.5)	NS

BMI, body mass index; NA, neutrophilic asthma; EA, eosinophilic asthma; PGA, paucigranulocytic asthma; MGA, mixed granulocytic asthma; ICS, inhaled corticosteroid; LABA, Long-acting β2-adrenergic receptor agonists; SABA, Short-acting β2 receptor agonist; COPD, chronic obstructive pulmonary disease; FEV1, forced expiratory volume in 1 s; FVC, forced vital capacity; AQLQ, Asthma Quality of Life Questionnaire; HADS, Hospital Anxiety and Depression scale; TCC, total cell count; NS, non-significant

Patients with severe asthma had clearly worse lung function, poorer asthma control (based on the ACT scores), and lower global AQLQ scores (especially in the “Activity” section) (*p* < 0.05). However, there was no significant difference in the HAD scores between the two groups. The severe asthma group had a higher proportion of patients who received their asthma diagnosis more than one year earlier and were treated by a specialist for more than 3 months compared to the non-severe asthma group (*p* < 0.05). In addition, a higher proportion of patients received ICS or ICS + LABA treatment in the severe asthma group (*p* < 0.001), and they were also treated with higher ICS doses (*p* < 0.001).

## Discussion

This cross-sectional study explored in detail the distribution of four different inflammatory phenotypes among asthma patients in Jilin Province in northeast China. Our study showed that the most common inflammatory phenotype was PGA (52.2%), followed by EA (38.3%), MGA (5.2%), and NA (4.3%). ICS treatment, older age, and smoking could increase sputum neutrophilic inflammation in patients with asthma [29–32]. The average age of patients with NA was the highest among the four asthma inflammatory phenotype groups in this study, and the prevalence of NA was higher among older patients. This confirmed previously published observations that sputum neutrophils in asthma increase with age [30]. In addition, smoking has also been shown to increase sputum neutrophilia [32]. In our study, smoking and overweight/obese patients exhibited a larger proportion of NA. However, the difference was not statistically significant (*p* > 0.05), possibly due to the inadequate sample size. Interestingly, the NA group received the highest average ICS treatment dose in the past year, and the neutrophil percentage in the sputum was positively correlated with the ICS treatment dose. However, since this is a cross-sectional study, it is difficult to conclude that ICS treatment increases sputum neutrophilia or that patients with sputum neutrophilia require higher doses of ICS. A previously published study suggested that the former might be the case as ICS can inhibit neutrophil apoptosis [33].

Notably, our results were significantly different from a previous report in Australia that showed that NA, EA, and PGA were present in 41%, 28%, and 31% of patients with asthma, respectively [8]. Moreover, our results were also distinctly different from a report from Beijing, China, which showed that the proportions of the four inflammatory phenotypes were 34.9% for NA, 34.9% for EA, 23.8% for MGA, and 6.3% for PA [7]. It is interesting that the proportion of NA was only 4.3% in our study, despite the fact that patients with asthma in our study had a higher average age (51 ± 14 vs 47 ± 17.6) and included a larger proportion of smokers (40.65 vs 19.6%), both factors that should have increased the proportion of NA [30, 32]. This may be related to the specific conditions in Northeast China.

Many reports have indicated that the abuse of antibiotics is a serious social problem in China [34]. Many regions in China have an extremely high frequency and intensity of antibiotics use [35–37]. The problem is especially severe in rural China, where individuals have low levels of knowledge on the use of antibiotics [38]. Most of the patients in our study came from remote rural areas in Northeast China, where the level of medical services is limited and the education level is low [39]. Research has shown that prudent antibiotic use during infancy might be beneficial for reducing the incidence of pediatric asthma by preserving the gut microbial community [40]. However, another study found that oral azithromycin treatment for 48 weeks could reduce asthma exacerbation in adults with persistent symptomatic asthma and improved quality of life [41]. Our results showed that antibiotic treatment significantly reduced the proportion of NA. Reasonable application of antibiotics might be a useful add-on therapy for asthma. Further, the abuse of antibiotics may be a reason for the lower proportion of NA in our region. In addition, there are significant differences in climate between the southern, northern, eastern, and western regions in China. Environmental factors might also affect the distribution of asthma inflammatory phenotypes.

Our study revealed that the disease severity is higher in patients with NA compared to the severity in those with EA. While 60% of patients with NA had severe asthma, the corresponding percentage was 18.2 for those with EA. Most patients with severe asthma in this study had been diagnosed more than one year earlier or were treated with a higher daily dose of ICS. This indicates an association between sputum neutrophilia and severe asthma. Similarly, a previous report also concluded that sputum neutrophil counts were correlated with more severe asthma phenotypes [11, 42]. However, in our study, the prevalence of severe asthma was low (19.1%). This might be one of the reasons for the low proportion of patients with NA found in our study.

Sputum eosinophilia is known to be correlated with uncontrolled asthma [43]. Although the difference was not significant (*p* > 0.05), the average ACQ6 and HAD scores were highest in the EA group, while the ACT score was the lowest in our study. Both the NA and EA groups had a higher proportion of patients who exhibited uncontrolled asthma. Similarly, the sputum eosinophil and neutrophil percentages as well as the proportions of EA and NA in patients with uncontrolled asthma were significantly higher in our study. A national cross-sectional study showed that asthma was largely underdiagnosed and undertreated in China, and the proportion of asthma respondents using ICS therapy was only 5.6%, which was much lower than those in European countries [44]. In our study, although 73% of patients exhibited uncontrolled asthma, only 47.1% of patients in the uncontrolled asthma group had been treated with ICS in the past year. In addition, fewer patients (51.2%) in the uncontrolled asthma group had been treated by a specialist for more than 3 months or received regular ICS treatment. A high number of patients in the uncontrolled asthma group persistently used SABA and low-dose ICS treatment. Thus, we speculate that irregular use of ICS and poor asthma control may be the main reasons for the higher proportion of EA in our study.

Exposure to diesel exhaust gas is known to significantly increase neutrophilic inflammation and reduce lung function in asthmatics [45, 46]. However, most of the patients in our study were from rural areas in Northeast China, where the main air pollutants are particulates from straw and coal burning [47], rather than traffic pollution. Importantly, the burning of agricultural residues is associated with various respiratory symptoms (cough, wheezing, chest tightness, shortness of breath) and respiratory diseases such as asthma [48–51]. An epidemiological study in our region showed that there is an association between ambient air pollution (mainly including straw and coal burning) and allergic rhinitis [52]. Therefore, straw and coal burning may be the reason for more eosinophilic inflammation in patients with asthma in our study. In addition, our study showed that the percentage of sputum macrophages in patients with PGA was significantly higher than that in healthy controls. We suspect that the air pollution in our region could have increased macrophages in the sputum of patients with asthma, thereby increasing the proportion of individuals with PGA in this region.

## Conclusions

The most common asthma inflammatory cell phenotype in Jilin Province, China is PGA, followed by EA, MGA, and NA. The low prevalence of NA in Jilin Province is strikingly different from the reported prevalence in other countries and other regions in China. Excessive antibiotic use in this region and irregular asthma ICS treatment may be the main reasons for the unique distribution of asthma inflammatory phenotypes.

## Data Availability

All data generated or analyzed during this study are included in this article.

## Abbreviations

EA: Eosinophilic asthma
NA: Neutrophilic asthma
MGA: Mixed granulocytic asthma
PGA: Paucigranulocytic asthma
FeNO: Fractional exhaled nitric oxide
ACQ6: 6-Item Asthma Control Questionnaire
ACT: Asthma Control Test Questionnaire
AQLQ: Asthma Quality of Life Questionnaire
HADS: Hospital Anxiety and Depression scale
GINA: Global Initiative for Asthma
FEV1: Predicted value of forced expiratory volume in 1 s
FVC: Forced vital capacity
ICS: Inhaled corticosteroid
COPD: Chronic obstructive pulmonary disease
LABA: Long-acting β2-adrenergic receptor agonists
SABA: Short-acting β2 receptor agonist

## References

[1] Barnes PJ (2017). Cellular and molecular mechanisms of asthma and COPD. Clin Sci (Lond).

[2] Fahy JV (2015). Type 2 inflammation in asthma–present in most, absent in many. Nat Rev Immunol.

[3] Simpson JL, Scott R, Boyle MJ, Gibson PG (2006). Inflammatory subtypes in asthma: assessment and identification using induced sputum. Respirology (Carlton, Vic).

[4] Brown HM (1958). Treatment of chronic asthma with prednisolone; significance of eosinophils in the sputum. Lancet (London, England).

[5] Schleich FN, Manise M, Sele J, Henket M, Seidel L, Louis R (2013). Distribution of sputum cellular phenotype in a large asthma cohort: predicting factors for eosinophilic vs neutrophilic inflammation. BMC Pulm Med.

[6] Taylor SL, Leong LEX, Choo JM, Wesselingh S, Yang IA, Upham JW, Reynolds PN, Hodge S, James AL, Jenkins C (2018). Inflammatory phenotypes in patients with severe asthma are associated with distinct airway microbiology. J Allergy Clin Immunol.

[7] Gai XY, Chang C, Wang J, Liang Y, Li MJ, Sun YC, He B, Yao WZ (2018). Airway inflammation and small airway wall remodeling in neutrophilic asthma. J Peking Univ Health Sci.

[8] Gibson PG (2009). Inflammatory phenotypes in adult asthma: clinical applications. Clin Respir J.

[9] Green RH, Brightling CE, Woltmann G, Parker D, Wardlaw AJ, Pavord ID (2002). Analysis of induced sputum in adults with asthma: identification of subgroup with isolated sputum neutrophilia and poor response to inhaled corticosteroids. Thorax.

[10] McGrath KW, Icitovic N, Boushey HA, Lazarus SC, Sutherland ER, Chinchilli VM, Fahy JV (2012). A large subgroup of mild-to-moderate asthma is persistently noneosinophilic. Am J Respir Crit Care Med.

[11] Choi JS, Jang AS, Park JS, Park SW, Paik SH, Park JS, Uh ST, Kim YH, Park CS (2012). Role of neutrophils in persistent airway obstruction due to refractory asthma. Respirology (Carlton, Vic).

[12] Rohde RA, Muller RA (2015). Air pollution in china: mapping of concentrations and sources. PLoS ONE.

[13] Yang G, Wang Y, Zeng Y, Gao GF, Liang X, Zhou M, Wan X, Yu S, Jiang Y, Naghavi M (2013). Rapid health transition in China, 1990–2010: findings from the Global Burden of Disease Study 2010. Lancet (London, England).

[14] Chen WW, Liu Y, Wu XW, Bao QY, Gao ZT, Zhang XL, Zhao HM, Zhang SC, Xiu AJ, Cheng TH (2019). Spatial and temporal characteristics of air quality and cause analysis of heavy pollution in northeast China. Huanjing kexue.

[15] Lin J, Wang W, Chen P, Zhou X, Wan H, Yin K, Ma L, Wu C, Li J, Liu C (2018). Prevalence and risk factors of asthma in mainland China: the CARE study. Respir Med.

[16] Wang G, Wang F, Gibson PG, Guo M, Zhang WJ, Gao P, Zhang HP, Harvey ES, Li H, Zhang J (2017). Severe and uncontrolled asthma in China: a cross-sectional survey from the Australasian Severe Asthma Network. J Thorac Dis.

[17] Global Initiative for Asthma. Global Strategy for asthma management and prevention. http://www.ginasthma.org. Accessed 2012 update.

[18] ATS/ERS recommendations for standardized procedures for the online and offline measurement of exhaled lower respiratory nitric oxide and nasal nitric oxide, 2005. Am J Respir Crit Care Med. 2005;171(8):912–30. 10.1164/rccm.200406-710ST 15817806

[19] Miller MR, Hankinson J, Brusasco V, Burgos F, Casaburi R, Coates A, Crapo R, Enright P, van der Grinten CP, Gustafsson P (2005). Standardisation of spirometry. Eur Respir J.

[20] Juniper EF, O'Byrne PM, Guyatt GH, Ferrie PJ, King DR (1999). Development and validation of a questionnaire to measure asthma control. Eur Respir J.

[21] Nathan RA, Sorkness CA, Kosinski M, Schatz M, Li JT, Marcus P, Murray JJ, Pendergraft TB (2004). Development of the asthma control test: a survey for assessing asthma control. J Allergy Clin Immunol.

[22] Juniper EF, Buist AS, Cox FM, Ferrie PJ, King DR (1999). Validation of a standardized version of the Asthma Quality of Life Questionnaire. Chest.

[23] Zigmond AS, Snaith RP (1983). The hospital anxiety and depression scale. Acta Psychiatr Scand.

[24] Global Initiative for Asthma. GINA. 2019. Cited 2019 Apr 29. http://ginasthma.org/.

[25] Global Initiative for Asthma. Global Strategy for asthma management and prevention. 2020. http://ginasthma.org/.

[26] Wittig HJ, Belloit J, De Fillippi I, Royal G (1980). Age-related serum immunoglobulin E levels in healthy subjects and in patients with allergic disease. J Allergy Clin Immunol.

[27] Gibson PG, Wlodarczyk JW, Hensley MJ, Gleeson M, Henry RL, Cripps AW, Clancy RL (1998). Epidemiological association of airway inflammation with asthma symptoms and airway hyperresponsiveness in childhood. Am J Respir Crit Care Med.

[28] Wong HH, Fahy JV (1997). Safety of one method of sputum induction in asthmatic subjects. Am J Respir Crit Care Med.

[29] Thomas RA, Green RH, Brightling CE, Birring SS, Parker D, Wardlaw AJ, Pavord ID (2004). The influence of age on induced sputum differential cell counts in normal subjects. Chest.

[30] Brooks CR, Gibson PG, Douwes J, Van Dalen CJ, Simpson JL (2013). Relationship between airway neutrophilia and ageing in asthmatics and non-asthmatics. Respirology (Carlton, Vic).

[31] Cowan DC, Cowan JO, Palmay R, Williamson A, Taylor DR (2010). Effects of steroid therapy on inflammatory cell subtypes in asthma. Thorax.

[32] Chalmers GW, MacLeod KJ, Thomson L, Little SA, McSharry C, Thomson NC (2001). Smoking and airway inflammation in patients with mild asthma. Chest.

[33] Saffar AS, Ashdown H, Gounni AS (2011). The molecular mechanisms of glucocorticoids-mediated neutrophil survival. Curr Drug Targets.

[34] Currie J, Lin W, Meng J (2014). Addressing antibiotic abuse in China: an experimental audit study. J Dev Econ.

[35] Wang Z, Zhang H, Han J, Xing H, Wu MC, Yang T (2017). Deadly sins of antibiotic abuse in China. Infect Control Hosp Epidemiol.

[36] Yan K, Xue M, Ye D, Yang C, Chang J, Jiang M, Zhao M, Zhang H, Fang Y (2018). Antibiotic prescribing practices in secondary and tertiary hospitals in Shaanxi province, western China, 2013–2015. PLoS ONE.

[37] Yin X, Song F, Gong Y, Tu X, Wang Y, Cao S, Liu J, Lu Z (2013). A systematic review of antibiotic utilization in China. J Antimicrob Chemother.

[38] Yu M, Zhao G, Stålsby Lundborg C, Zhu Y, Zhao Q, Xu B (2014). Knowledge, attitudes, and practices of parents in rural China on the use of antibiotics in children: a cross-sectional study. BMC Infect Dis.

[39] Dong H, Hao Y, Li D, Su Z, Li W, Shi B, Gao P (2020). Risk factors for acute exacerbation of chronic obstructive pulmonary disease in industrial regions of China: a multicenter cross-sectional study. Int J Chronic Obstr Pulm Dis.

[40] Patrick DM, Sbihi H, Dai DLY, Al Mamun A, Rasali D, Rose C, Marra F, Boutin RCT, Petersen C, Stiemsma LT (2020). Decreasing antibiotic use, the gut microbiota, and asthma incidence in children: evidence from population-based and prospective cohort studies. Lancet Respir Med.

[41] Gibson PG, Yang IA, Upham JW, Reynolds PN, Hodge S, James AL, Jenkins C, Peters MJ, Marks GB, Baraket M (2017). Effect of azithromycin on asthma exacerbations and quality of life in adults with persistent uncontrolled asthma (AMAZES): a randomised, double-blind, placebo-controlled trial. Lancet (London, England).

[42] Moore WC, Hastie AT, Li X, Li H, Busse WW, Jarjour NN, Wenzel SE, Peters SP, Meyers DA, Bleecker ER (2014). Sputum neutrophil counts are associated with more severe asthma phenotypes using cluster analysis. J Allergy Clin Immunol.

[43] Gao J, Chen Z, Jie X, Ye R, Wu F (2018). Both fractional exhaled nitric oxide and sputum eosinophil were associated with uncontrolled asthma. J Asthma Allergy.

[44] Huang K, Yang T, Xu J, Yang L, Zhao J, Zhang X, Bai C, Kang J, Ran P, Shen H (2019). Prevalence, risk factors, and management of asthma in China: a national cross-sectional study. Lancet (London, England).

[45] McCreanor J, Cullinan P, Nieuwenhuijsen MJ, Stewart-Evans J, Malliarou E, Jarup L, Harrington R, Svartengren M, Han IK, Ohman-Strickland P (2007). Respiratory effects of exposure to diesel traffic in persons with asthma. N Engl J Med.

[46] Holgate ST, Sandström T, Frew AJ, Stenfors N, Nördenhall C, Salvi S, Blomberg A, Helleday R, Söderberg M (2003). Health effects of acute exposure to air pollution. Part I: healthy and asthmatic subjects exposed to diesel exhaust. Res Rep (Health Effects Inst).

[47] Wen X, Chen W, Chen B, Yang C, Tu G, Cheng T (2020). Does the prohibition on open burning of straw mitigate air pollution? An empirical study in Jilin Province of China in the post-harvest season. J Environ Manag.

[48] Long W, Tate RB, Neuman M, Manfreda J, Becker AB, Anthonisen NR (1998). Respiratory symptoms in a susceptible population due to burning of agricultural residue. Chest.

[49] Youssouf H, Liousse C, Roblou L, Assamoi EM, Salonen RO, Maesano C, Banerjee S, Annesi-Maesano I (2014). Non-accidental health impacts of wildfire smoke. Int J Environ Res Public Health.

[50] Prasad R, Singh A, Garg R, Giridhar GB (2012). Biomass fuel exposure and respiratory diseases in India. Biosci Trends.

[51] Torigoe K, Hasegawa S, Numata O, Yazaki S, Matsunaga M, Boku N, Hiura M, Ino H (2000). Influence of emission from rice straw burning on bronchial asthma in children. Pediatr Int.

[52] Teng B, Zhang X, Yi C, Zhang Y, Ye S, Wang Y, Tong DQ, Lu B. The association between ambient air pollution and allergic rhinitis: further epidemiological evidence from Changchun, northeastern China. Int J Environ Res Public Health. 2017;14(3). 10.3390/ijerph14030226 PMC5369062 28241509

